# Granulosa cell-derived induced pluripotent stem cells exhibit pro-trophoblastic differentiation potential

**DOI:** 10.1186/s13287-015-0005-5

**Published:** 2015-02-27

**Authors:** Ching-Yu Chuang, Mei-Chi Huang, Hsin-Fu Chen, Li-Hui Tseng, Chun-Ying Yu, Lee Stone, Hsiang-Po Huang, Hong-Nerng Ho, Hung-Chih Kuo

**Affiliations:** Genomics Research Center, Academia Sinica, Taipei, 115 Taiwan; Institute of Cellular and Organismic Biology, Academia Sinica, 128 Academia Road, Sec. 2, Nankang District, Taipei, 115 Taiwan; Graduate Institute of Clinical Genomics, College of Medicine, National Taiwan University, Taipei, 115 Taiwan; Department of Obstetrics and Gynecology, Division of Reproductive Endocrinology and Infertility, National Taiwan University and Hospital, Taipei, 115 Taiwan; Department of Medical Genetics, National Taiwan University Hospital, Taipei, 115 Taiwan

## Abstract

**Introduction:**

Human induced pluripotent stem cells (hiPSCs) have been derived from various somatic cell types. Granulosa cells, a group of cells which surround oocytes and are obtained from the (normally discarded) retrieved egg follicles of women undergoing infertility treatment, are a possible cell source for induced pluripotent stem cell (iPSC) generation. Here, we explored the possibility of using human granulosa cells as a donor cell type for iPSC reprogramming, and compared granulosa cell-derived iPSCs (iGRAs) with those derived from other cell sources, to determine the potential ability of iGRA differentiation.

**Methods:**

Granulosa cells were collected from egg follicles retrieved from women undergoing infertility treatment. After short-term culture, the granulosa cells derived from different patients were mixed in culture, and infected with retroviruses encoding reprogramming factors. The resulting iPSC clones were selected and subjected to microsatellite DNA analysis to determine their parental origin. IGRAs were subjected to RT-PCR, immunofluorescence staining, and *in vitro* and *in vivo* differentiation assays to further establish their pluripotent characteristics.

**Results:**

Microsatellite DNA analysis was used to demonstrate that hiPSCs with different parental origins can be simultaneously reprogrammed by retroviral transfection of a mixed human granulosa cell population obtained from multiple individuals. The iGRAs resemble human embryonic stem cells (hESCs) in many respects, including morphological traits, growth requirements, gene and marker expression profiles, and *in vitro* and *in vivo* developmental propensities. We also demonstrate that the iGRAs express low levels of NLRP2, and differentiating iGRAs possess a biased differentiation potential toward the trophoblastic lineage. Although NLRP2 knockdown in hESCs promotes trophoblastic differentiation of differentiating hESCs, it does not result in exit from pluripotency. These results imply that NLRP2 may play a role in regulating the trophoblastic differentiation of human pluripotent stem cells.

**Conclusions:**

These findings provide a means of generating iPSCs from multiple granulosa cell populations with different parental origins. The ability to generate iPSCs from granulosa cells not only enables modeling of infertility-associated disease, but also provides a means of identifying potential clinical interventions through iPSC-based drug screening.

**Electronic supplementary material:**

The online version of this article (doi:10.1186/s13287-015-0005-5) contains supplementary material, which is available to authorized users.

## Introduction

Human induced pluripotent stem cells (hiPSCs) are generated from somatic cells by overexpression of a panel of transcription factors, including OCT4, SOX2, KLF4, and c-MYC [1]. The resulting hiPSCs exhibit the typical characteristics of human embryonic stem cells (hESCs); not only do they express surface and pluripotency-related markers, but they are also able to give rise to cell types representing all three embryonic germ layers, as demonstrated by both *in vitro* differentiation and *in vivo* teratoma formation analysis. Induced pluripotent stem cell (iPSC) technology therefore provides an easy and efficient means of generating embryonic stem cell (ESC)-like cells from any individual. The availability of iPSCs circumvents the ethical disputes and immunological problems arising from the use of hESCs, thereby opening up new possibilities for disease modeling and stem cell-based therapies.

At the time of writing, fibroblasts are the most common donor source for iPSC generation; however, a variety of alternative cell types have also been used for the derivation of iPSC lines, on account of their availability or ease of reprogramming. One such example is peripheral blood cells, which are widely used because of the ease with which they can be obtained from patients and because of their ability to be reprogrammed without the need for extensive cell culture [2,3]. Human keratinocytes [4], neural stem cells [5,6], and cord blood CD133^+^ cells [7] have a higher reprogramming efficiency than human fibroblasts and/or require fewer transcription factors for reprogramming; this is believed to be due to their expression of pluripotent genes, or possession of an epigenomic regulatory pattern that is closer to ESCs than that of fibroblasts. Previous studies indicated that differences between the origins of cell types influence reprogramming efficiency, as well as the *in vitro* differentiation potential of iPSCs. For example, analysis of early-passage iPSCs (derived from mouse fibroblasts, and hematopoietic and myogenic cells) indicated that these cells possess different transcriptional and epigenetic profiles, which results in distinctive *in vitro* differentiation potentials [8]. Therefore, it has become apparent that selection of the donor cell type for generation of iPSCs is a critical issue because the parental cell type affects the efficiency of reprogramming, the requirements for type and quality of ectopic transcription factors, the *in vitro* and *in vivo* developmental propensities, and the epigenetic memory of the resulting iPSCs.

Human granulosa cells are crucial for the growth and development of oocytes during ovarian folliculogenesis. These cells not only secrete the hormones required for ovulation and endometrial proliferation, but their normal function is also required for avoiding disorders of the human ovary, including polycystic ovary syndrome [9], premature ovarian failure [10], and granulosa cell tumors [11]. Although granulosa cells are important for female reproduction, the understanding of their involvement in ovarian function and dysfunction is limited, mostly due to the difficulty in generating a suitable model for *in vitro* study. With advances in assisted reproductive techniques and *in vitro* culture methods, human granulosa cells have become available for use in such studies; granulosa cells can be retrieved from infertile women, and prolonged culture of these cells *in vitro* can be achieved by adding leukemia inhibitory factor [12]. Thus, we explored the possibility of using human granulosa cells as a donor cell type for iPSC reprogramming, and compared granulosa cell-derived induced pluripotent stem cells (iGRAs) with those derived from other cell sources, to determine the potential suitability of iGRAs for the study of female reproductive disease.

In this study, we demonstrate that hiPSC clones can be simultaneously generated from human granulosa cells with different parental origins. Although human iGRAs resembled hESCs in terms of their pluripotent characteristics and *in vivo*/*in vitro* differentiation potential, iPSCs derived from granulosa cells exhibited a greater propensity to differentiate to the trophoblast lineage as compared with hESCs and other iPSC lines. In addition, the expression of NLRP2 was found to be generally lower in human iGRAs than in other cell lines, and this was confirmed to be associated with preferential differentiation into trophoblasts.

## Materials and methods

### Ovarian stimulation and collection of human granulosa cells

The ovarian stimulation protocol involved combined use of short flare-up gonadotropin-releasing hormone agonist and recombinant follicle-stimulating hormone (225 IU/day for 10 days, GONAL-f PEN; Merck Serono, Merck KGaA, Darmstadt, Germany). When at least two follicles reached a size of 18 mm, human chorionic gonadotropin (Ovidrel; Merck Serono, Merck KGaA, Darmstadt, Germany) was used to trigger final maturation of the oocytes. Transvaginal oocyte retrieval was then performed approximately 35 hours after injection of human chorionic gonadotropin. Human granulosa cells were obtained from the pooled follicular fluid aspirates from patients. Granulosa cells were enriched in accordance with a recently published protocol [13].

### Cell culture

Human granulosa cells from three Asian females and foreskin fibroblasts from a 28-year-old male were obtained with written informed consent from tissue donors, in accordance with the protocol approved by the Research Ethics Committee of National Taiwan University Hospital and the Internal Research Board of Academia Sinica. Human granulosa cells and foreskin fibroblasts were grown and expanded in Dulbecco’s modified Eagle’s medium (Invitrogen, Carlsbad, CA, USA) containing 15% fetal bovine serum (Invitrogen), 2 mM l-glutamine (Invitrogen), 0.1 mM nonessential amino acids (Invitrogen), and 1% penicillin/streptomycin (Invitrogen). Human follicle dermal papilla cells were purchased from PromoCell Inc. (Heidelberg Germany) and maintained in Human Follicle Dermal Papilla Cell Growth Medium (PromoCell Inc.) according to the manufacturer’s protocol. These cells were subjected to iPSC induction within four passages after receipt.

The hiPSCs and hESCs, H9 (46, XX; WiCell Research Institute Inc., Madison, WI, USA) and NTU1 (46, XX; National Taiwan University and Hospital, Taipei, Taiwan), were grown on mitotically-inactivated mouse embryonic fibroblasts in Dulbecco’s modified Eagle’s medium/F12 (Invitrogen) with 20% Knockout Serum Replacement (Invitrogen), and basic fibroblast growth factor (4 ng/ml; Sigma, St. Louis, MO, USA). Passage of hiPSCs and hESCs was performed every 5 to 7 days by manual splitting.

### Retroviral infection and iPSC generation

Derivation of human iPSCs was performed as described previously [14]. All plasmids for generating iPSCs were purchased from Addgene (Cambridge, MA, USA); these plasmids included pMXs-hOCT3/4 (Addgene 17217), pMXs-hSOX2 (Addgene 17218), pMXs-hKLF4 (Addgene 17219), and pMXs-hc-MYC (Addgene 17220).

For granulosa cells, four consecutive transductions were performed. Six days after the first transduction, fibroblasts and papilla cells were trypsinized and reseeded at 5 × 10^4^ cells per 100 mm dish on mouse embryonic fibroblast feeders. Granulosa cells were trypsinized and replated at 1 × 10^5^ cells per 100 mm dish on mouse embryonic fibroblast feeders 8 days after the first transduction. On the next day, the media were replaced with hESC media, as described above. Approximately 30 days after transduction, colonies were picked manually and transferred into 0.5 ml hESC media in 24-well plates, before being scaled up.

### Characterization of human iPSCs

Genomic DNA and RNA were extracted from reprogrammed clones with the DNeasy Mini Kit and the RNeasy Mini Kit separately (Qiagen, Hilden, Germany). Integration of retroviral transgenes was examined by PCR analysis with specific primers; in addition, the expression of endogenous genes and viral transgenes (OCT4, SOX2, KLF4, c-MYC, and other pluripotency genes) of reprogrammed cells was assessed by RT-PCR with specific primers [1].

### Generation of constitutive knockdown hESCs

The shNLRP2 and shLuc constructs were obtained from the National RNAi Core Facility Platform (National Science Council, Taipei, Taiwan). The target sequences for shNLRP2 constructs were as follows: shNLRP2-128236, GCTGAATCACATAGGAGTTAA; shNLRP2-130989, CCAGGTTATGGCTGAGAGATA; and shNLRP2-129801, CTCAGGGATAATGAGTTCATT. Lentiviral production and hESC infection were performed in accordance with a procedure described previously [15].

### *In vitro* random and trophoblastic differentiation of iPSCs and hESCs

The protocols for embryonic body formation and random differentiation were described previously [16]. Trophoblastic differentiation was induced using the protocol of Xu and colleagues [17], and recombinant human BMP4 (R&D, Minneapolis, MN, USA) was added at a concentration of 20 ng/ml.

### RNA isolation, reverse transcription, PCR, and quantitative PCR

Total RNA was extracted from hESCs, iPSCs, and their differentiated cells, and then treated with DNase I (Qiagen). Total RNA was converted to cDNA using the High Capacity cDNA Reverse Transcriptase Kit (Applied Biosystems, Foster City, CA, USA). PCR was performed with the Platinum Taq DNA polymerase kit (Invitrogen) according to the manufacturer’s instructions. Quantitative PCR was performed with the SYBR FAST ABI Prism qPCR Kit (KAPA, Wilmington, MA, USA), and was analyzed with the 7900 real-time PCR system (Applied Biosystems). Results were normalized using GAPDH, and analyzed based on the relative quantification (ΔΔ-Ct method). Primer sequences are shown in Additional file 1.

### Immunofluorescence staining, western blot analysis, and teratoma formation

Immunofluorescence (IF) staining, western blot analysis, and teratoma formation were performed as described previously [16]. Primary antibodies against the following proteins were used in this study: OCT4 (1:200), SSEA4 (1:200), TRA-1-60 (1:200), TG30 (1:200), HESCA-1 (1:200), HESCA-2 (1:200), MAP2 (1:500), CDX2 (1:200), and EOMES (1:200; Millipore, Temecula, CA, USA), PAX6 (1:50; DSHB, Iowa City, IA, USA), AFP (1:500; Dako, Glostrup, Denmark), SOX17 (1:200) and CG-α (1:500; R&D), GATA4 (1:200) and GATA2 (1:500; Santa Cruz, Dallas, TX, USA), a-SMA (1:500) and β-actin (1:800; Sigma), and CG-β (1:500) and NLRP2 (1:1,000; Abcam, Cambridge, UK). The following secondary antibodies were used: goat anti-mouse, rabbit Cy3 (1:500; Jackson ImmunoResearch laboratories, West Grove, PA, USA); and goat anti-mouse, rabbit 488 (1:200; Invitrogen).

### Microsatellite assays for human parentage testing

Genomic DNA was extracted from 13 selected iGRAs, and identity analysis was performed using the AmpFlSTR Identifiler kit (Applied Biosystems), according to the manufacturer’s instructions. Fifteen microsatellite loci (D8S1179 on chromosome 8, D21S11 at 21q11.2, D7S820 at 7q, CSF1PO at 5q33.3-34 labeled with 6-FAM, D3S1358 at chromosome 3p, THO1 at 11p15.5, D13S317 at 13q22-31, D16S539 at 16q24-qter, D2S1338 at 2q35-37.1 labeled with VIC, D19S433 at 19q12-13.1, vWA at 12p12-pter, TPOX at 2p25.3, D18S51 at 18q21.3 labeled with NED, D5S818 at 5q21-31, and FGA at 4q28 labeled with PET) and the amelogenin loci on the X (p22.1-22.3) and Y (p11.2) chromosomes were analyzed.

### Microarray analysis

Two micrograms of total RNA purified by Trizol (Invitrogen) were used to generate biotin-labeled cRNA probes, which were hybridized to the Affymetrix Human Genome U133 plus 2.0 array (Affymetrix, Santa Clara, CA, USA) by the Affymetrix gene expression service laboratory at Academia Sinica (Taipei, Taiwan). Chips were scanned using the Affymetrix GeneChip Scanner 7G, and expression profiles were analyzed with GeneSpring XI software (Agilent, Santa Clara, CA, USA). Two biological replicates were performed for each cell line. Raw data were normalized with Robust Multichip Average, and weakly expressed signals (means <20% of total samples) were excluded. The raw microarray data are available through the Gene Expression Omnibus [GEO:GSE28406, GEO:GSE19964].

### Immunoassays of placental hormones

The media from trophoblastic differentiation were collected on days 0, 2, 4, 7, 12, and 14, and were examined for the presence of β-HCG (IBL international GmbH, Hamburg, Germany), estradiol, and progesterone (Cayman Chemical Company, Ann Arbor, MI, USA) using enzyme immunoassays.

## Results

### A one-pot method to simultaneously derive iPSC lines from granulosa cells obtained from multiple donors

**Figure 1:**
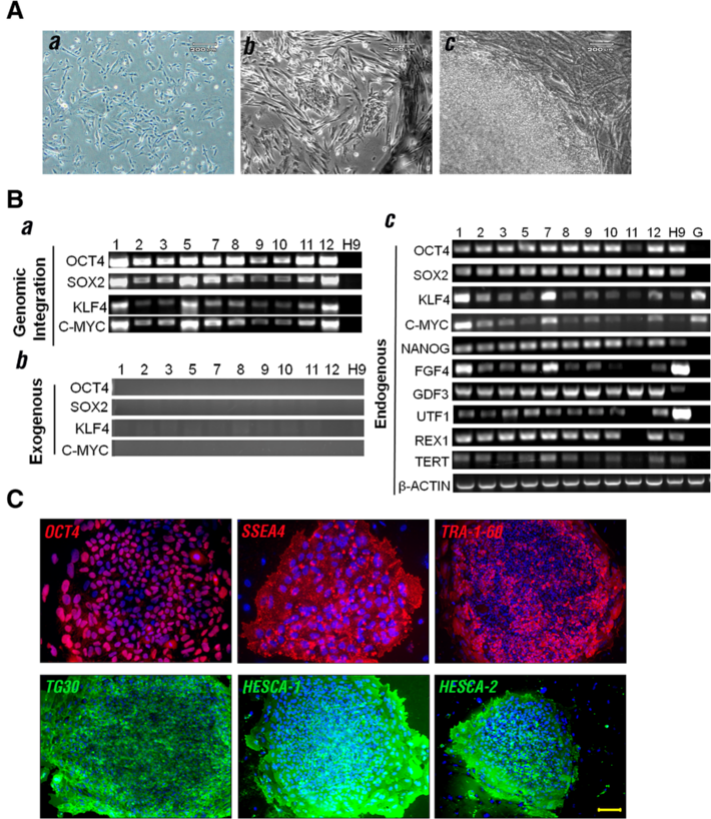
**Derivation and characterization of induced pluripotent stem cells derived from human granulosa cells. (A)** Reprogramming of induced pluripotent stem cell (iPSC) lines: **(a)** parental granulosa cells at early passage (P1), **(b)** early reprogrammed cell colonies, and **(c)** human embryonic stem cell (hESC)-like colonies prior to being manually picked. **(B)** Genetic analysis of reprogrammed cell lines: **(a)** genomic PCR was used to reveal integration of all four retroviral transgenes, and **(b)** RT-PCR was used to demonstrate inactivation of exogenous viral transgenes and **(c)** expression of endogenous pluripotency genes. H9 hESCs (H9) and human granulosa cells (G) were used as controls for PCR and RT-PCR reactions. **(C)** Immunofluorescence analysis of pluripotency hESC markers (OCT4, SSEA4, TRA-1-60, TG30, HESCA-1, and HESCA-1) in a representative human granulosa cell-derived iPSC line (iGRA1). Scale bars = 200 μm (A,a,b,c) and 50 μm **(C)**.

To generate iPSCs, we obtained human granulosa cells from three female donors. Since the granulosa cell population of each donor is small, we mixed the cells from each individual and cultured them together. These cells were then subjected to retroviral infection with vectors containing human OCT4, SOX2, KLF4, and c-MYC cDNA during their early passages (P1 to P4) (Figure 1A,a). On day 20, around 100 early reprogramming cell colonies were detected within the initial 2 × 10^5^ granulosa cells (Figure 1A,b and Table 1). A total of 30 iPSC clones were manually picked based on their morphological traits at 30 days post viral infection (Figure 1A,c), and 24 of them were used to successfully establish iPSC lines (Table 1). Genomic PCR and RT-PCR using primers specific for the retroviral transcripts [1] were performed to reveal integration and silencing of exogenic OCT4, SOX2, KLF4, and c-MYC genes in 10 of the 24 clones (Figure 1B,a,b). The pluripotent potential of the selected iPSC lines was further demonstrated by the expression of endogenous pluripotency-associated genes and hESC markers (Figure 1B,c,C).

**Table 1:** **Summary of human induced pluripotent stem cell generation**

**Parental cell type**	**Number of parental cells seeded**	**Number of early reprogramming colonies**	**Number of established iPSCs**
Foreskin	5 × 10^4^	>300	48
Follicle dermal papilla cells	5 × 10^4^	>300	5
Granulosa cells	2 × 10^5^	<100	24

iPSC, induced pluripotent stem cell.

**Figure 2:**
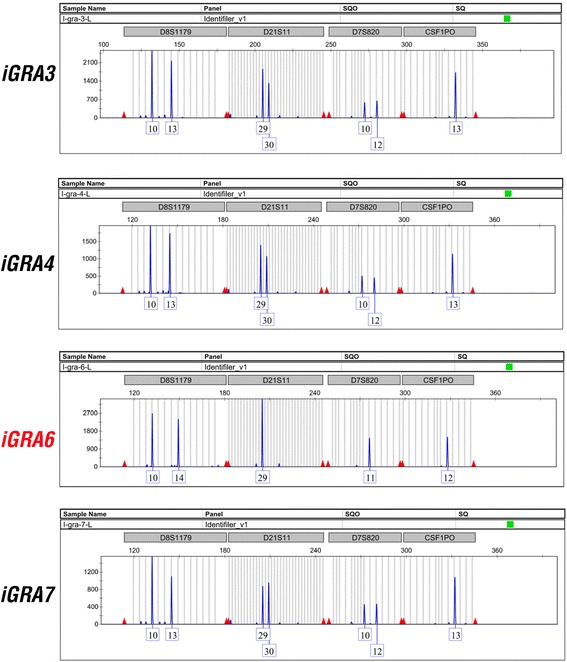
**Microsatellite marker analysis for human granulosa cell-derived induced pluripotent stem cells.** Four representative induced pluripotent stem cell clones and four representative microsatellite loci are shown.

To identify the original donors of the derived iPSC lines, we performed a DNA microsatellite assay to determine the parentage of each clone. A total of 15 microsatellite loci and one amelogenin locus on the sex chromosomes were analyzed for each of the selected 13 iGRAs. Such analyses revealed that one donor resulted in 12 of the 13 iPSC lines, a second donor resulted in a single line (iGRA6), while the third donor was not represented in any of the established iPSC lines (Figure 2 and Additional file 2). These results demonstrate that it is possible to simultaneously derive multiple iPSC clones from mixed granulosa cell samples derived from multiple individuals.

### Granulosa cell-derived iPSCs exhibit pluripotent characteristics both *in vitro* and *in vivo*

**Figure 3:**
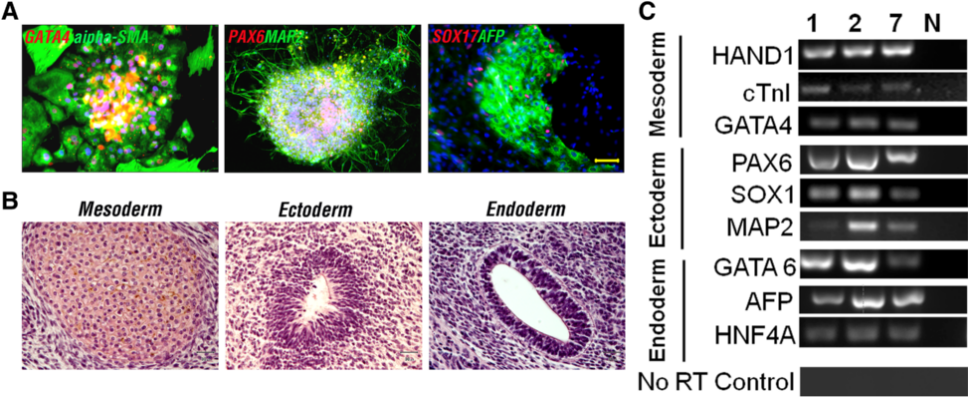
***In vitro*** **and** ***in vivo*** **differentiation of human granulosa cell-derived induced pluripotent stem cells. (A)** Immunofluorescence staining against three germ layer markers in a representative induced pluripotent stem cell line (iGRA1) following *in vitro* differentiation. **(B)** Hematoxylin and eosin staining of teratoma derived from a representative iGRA line (iGRA1). **(C)** RT-PCR analysis of expression of genes associated with three germ layers in *in vitro* differentiated iGRAs. 1, 2 and 7, iGRA1, iGRA2, and iGRA7, respectively. N, RNA transcripts untreated with reverse transcriptase (no RT control) served as a negative control for genomic DNA contamination. Scale bars = 50 μm **(A)** and 30 μm **(B)**.

To investigate their developmental propensity, three iGRAs (iGRA1, iGRA2, and iGRA7) were subjected to both *in vitro* and *in vivo* differentiation procedures. For *in vitro* differentiation, embryonic body was generated to allow spontaneous differentiation. At around 20 days, differentiated cells expressing markers of mesoderm (GATA4 and α-SMA), ectoderm (PAX6 and MAP2), and endoderm (SOX17 and AFP) were identified by IF staining (Figure 3A). RT-PCR was used to confirm the IF staining data, by detecting expression of genes related to mesoderm (Hand1, cTnI, and GATA4), ectoderm (PAX6, SOX1, and MAP2), and endoderm (GATA6, AFP, and HNF4A) in the *in vitro* differentiated iPSCs at around day 20 (Figure 3C). In addition, teratoma formation was assessed to determine developmental potential *in vivo*. Combined histological and immunohistochemical analysis of iGRA-induced teratomas revealed that the cells had differentiated into cell types representing all three embryonic germ layers (Figure 3B).

**Figure 4:**
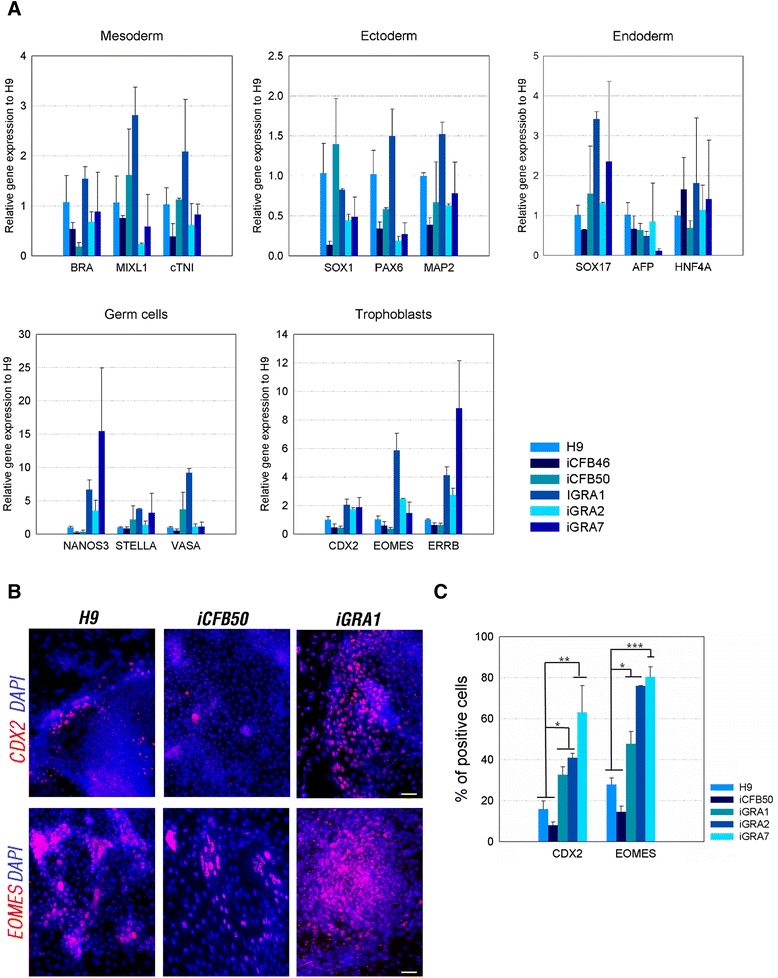
**Comparison of the** ***in vitro*** **differentiation potential of human embryonic stem cell and induced pluripotent stem cell lines. (A)** Quantitative PCR analysis of genes related to three germ layers, germ cells, and trophoblasts in human embryonic stem cells (hESCs) and induced pluripotent stem cells (iPSCs) following *in vitro* differentiation. The iPSCs (iCFB46, iCFB50, iGRA1, iGRA2, and iGRA7) and hESCs (H9) were examined at day 20 of *in vitro* differentiation. Relative gene expression was first normalized to that of GAPDH, and then represented as the fold change relative to H9. Values represent the mean ± standard deviation (SD) (*n* = 3). Statistical analysis for lineage-specific gene expression is presented in Additional file 3. **(B)** Immunofluorescence staining against CDX2 and EOMES on day 7 of trophoblastic differentiation of representative H9, iCFB50, and iGRA1 cells. Scale bars = 50 μm. **(C)** Quantitative analysis of CDX2 and EOMES positive cells from day 7 of trophoblastic differentiation of H9, iCFB50, and iGRA1, iGRA2, and iGRA7. Approximately 600 cells were counted for each experiment. Values are means ± SD (*n* = 3). *P <0.05, **P <0.01, ***P <0.001.

To further explore the differentiation potential of iGRAs, quantitative PCR was used to quantify the expression of lineage-specific genes. The expression patterns of mesoderm, ectoderm, endoderm, and germ cell genes were not consistent between H9 hESCs, foreskin-derived iPSCs (iCFB46 and iCFB50), and three iGRA lines with the same parental origin (Figure 4A and Additional file 3). The differentiation potential of these pluripotent cell lines seemed to vary between different iPSC clones. Notably, the expression levels of early trophoblast-related genes, such as CDX2, EOMES, and ERRB, were higher in the iGRA clones than in hESCs and foreskin-derived iPSCs (Figure 4A and Additional file 3). Next, we used antibodies against trophoblastic antigens (including CDX2 and EOMES) to perform IF staining on cells (derived from hESC (H9), hiPSC (iCFB50), and iGRA1, iGRA2, and iGRA7) undergoing trophoblastic differentiation. Our quantitative analysis revealed a significantly higher number of CDX2-positive or EOMES-positive cells for differentiated iGRA1, iGRA2, and iGRA7 cells than for H9 and iCFB50 cells (Figure 4B,C). These results suggest that iGRAs may be prone to spontaneously differentiate into trophoblastic lineages *in vitro*.

### NLRP2 was expressed at low levels in human granulosa cell-derived iPSCs

**Figure 5:**
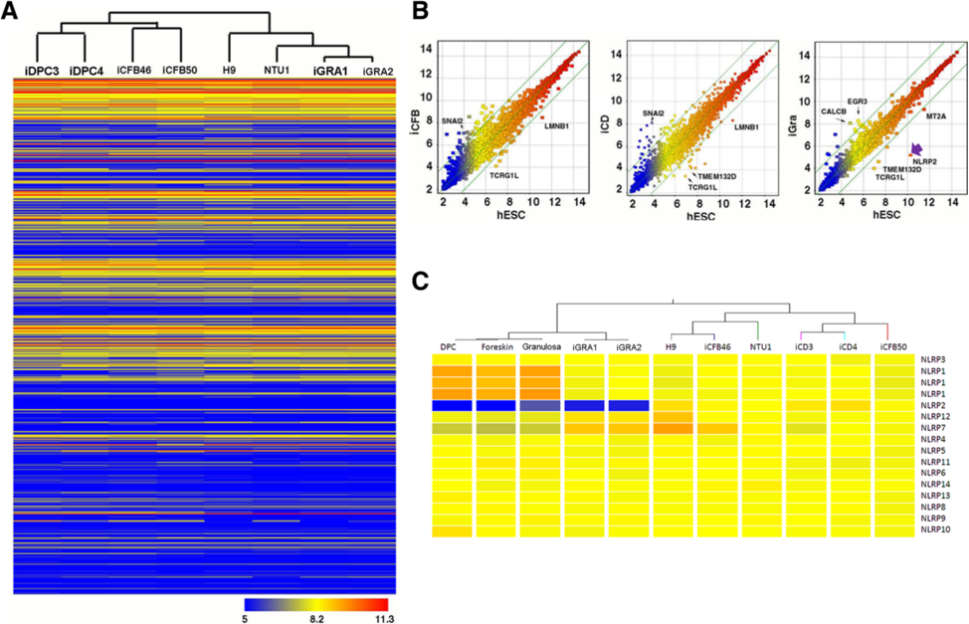
**Microarray-based analysis of global gene expression in multiple human embryonic stem cell and induced pluripotent stem cell clones. (A)** Hierarchical clustering of global expression profiles of human embryonic stem cells (hESCs) (H9 and NTU1) and induced pluripotent stem cells (iPSCs) (iCFB46, iCFB50, iDPC3, iDPC4, iGRA1, and iGRA2). **(B)** Scatter plots of relative gene expression between hESCs and iPSCs (hESC to iCFB, hESC to iDPC, and hESC to iGRA). Each dot represents the averaged expression level of one gene in different samples. Green lines, fourfold changes between two samples; purple arrow, low expression level of the NLRP2 gene in iGRAs as compared with hESCs. **(C)** Heat map showing the expression levels of NLRP family members in hESCs, iPSCs, and their parental cells. Sex-related genes were excluded during microarray analysis to avoid sex bias between hESCs (H9 and NTU1 are both 46, XX) and iPSCs.

The results described above indicate that iGRAs preferentially differentiate towards the trophoblastic lineage; we proceeded to investigate the possible mechanism underlining this observation by comparing the global gene-expression profiles of the parental cells of iPSCs, hESCs (H9 and NTU1), foreskin-derived iPSCs (iCFB46 and iCFB50), follicle dermal papilla cell-derived iPSCs (iDPC3 and iDPC4), and iGRA1 and iGRA2 using cDNA microarray analysis. Pearson correlation analysis revealed that the transcriptional profiles of all iPSC lines were similar to those of hESCs, and different from those of their parental cells (Additional file 4). Comparison of the global gene expression patterns for all of the pluripotent stem cell lines revealed that iGRAs clustered more closely with hESCs than with other lines (Figure 5A).

**Figure 6:**
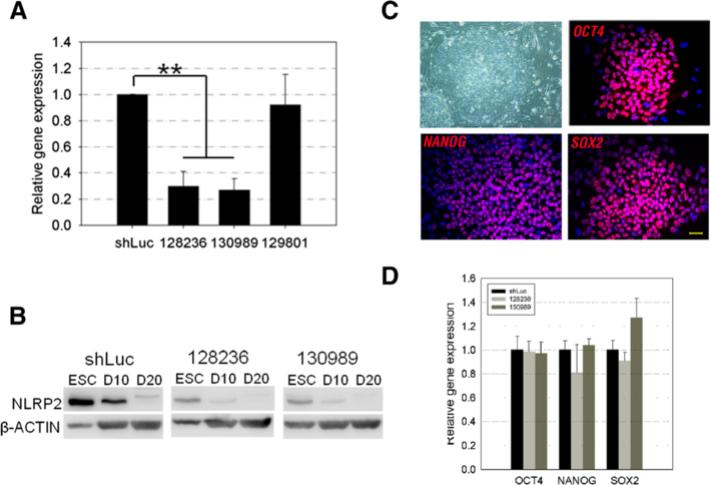
**Constitutive knockdown of NLRP2 in H9 human embryonic stem cells. (A)** NLRP2 gene expression in shLuc (control) and knockdown (KD) NLRP2 (128236, 130989, and 129801) H9 embryonic stem cells (ESCs), as determined by quantitative PCR. Values are mean ± standard deviation (SD) (*n* = 3). *P <0.01. **(B)** NLRP2 protein expression in shLuc and KD NLRP2 (128236 and 130989) H9 ESCs before (ESC) and during *in vitro* differentiation (days 10 (D10) and 20 (D20)), as determined by western blot. **(C)** Bright-field imaging and immunofluorescence staining of KD NLRP2 H9 ESCs. Scale bar = 50 μm. **(D)** Expression of pluripotent genes in shLuc and KD NLRP2 (128236 and 130989) H9 ESCs by quantitative PCR. Relative gene expression was first normalized to that of GAPDH, and then presented as the fold change relative to that in shLuc H9 ESCs. Values are mean ± SD (*n* = 3).

However, expression levels of NLRP2, a gene required for preimplantation development of human and mouse [18,19], were significantly lower in all tested iGRAs than in hESCs (Figure 5B,C and Additional file 5). Notably, the expression level of NLRP2 in iPSC parental cells is low, and is significantly increased after pluripotency reprogramming (Figure 5C and Additional file 5). Furthermore, its expression level decreased significantly in hESCs after *in vitro* differentiation (Figure 6B and Additional file 6). The results obtained with quantitative PCR are consistent with the microarray data (Additional file 5). Thus, we have confirmed that iGRAs exhibit relatively low expression of NLRP2 and preferential differentiation into trophoblasts. We therefore hypothesize that the low expression of NLRP2 may result in the preferential trophoblastic differentiation potential of iGRAs.

### Knockdown of NLRP2 expression promotes trophoblastic differentiation in hESCs

To confirm whether low expression of NLRP2 results in the preferential trophoblastic differentiation potential of iGRAs, we used lentiviral-based RNA interference to specifically knockdown (KD) NLRP2 mRNA in H9 hESCs. H9 hESCs were transfected with the shLuc control, or one of three KD NLRP2 variants (−128236, −130989, and −129801). KD NLRP2 128236 and 130989 caused a significant reduction of NLRP2 expression, as evidenced by quantitative PCR and western blotting analysis (Figure 6A,B). However, we did not observe significant morphological changes in NLRP2 KD hESCs, or changes in the expression levels (mRNA and protein) of pluripotency-associated genes/markers (OCT4, NANOG, and SOX2) as compared with control hESCs transfected with shLuc (Figure 6C,D); these findings suggest that disruption of NLRP2 expression in hESCs does not affect pluripotency maintenance.

**Figure 7:**
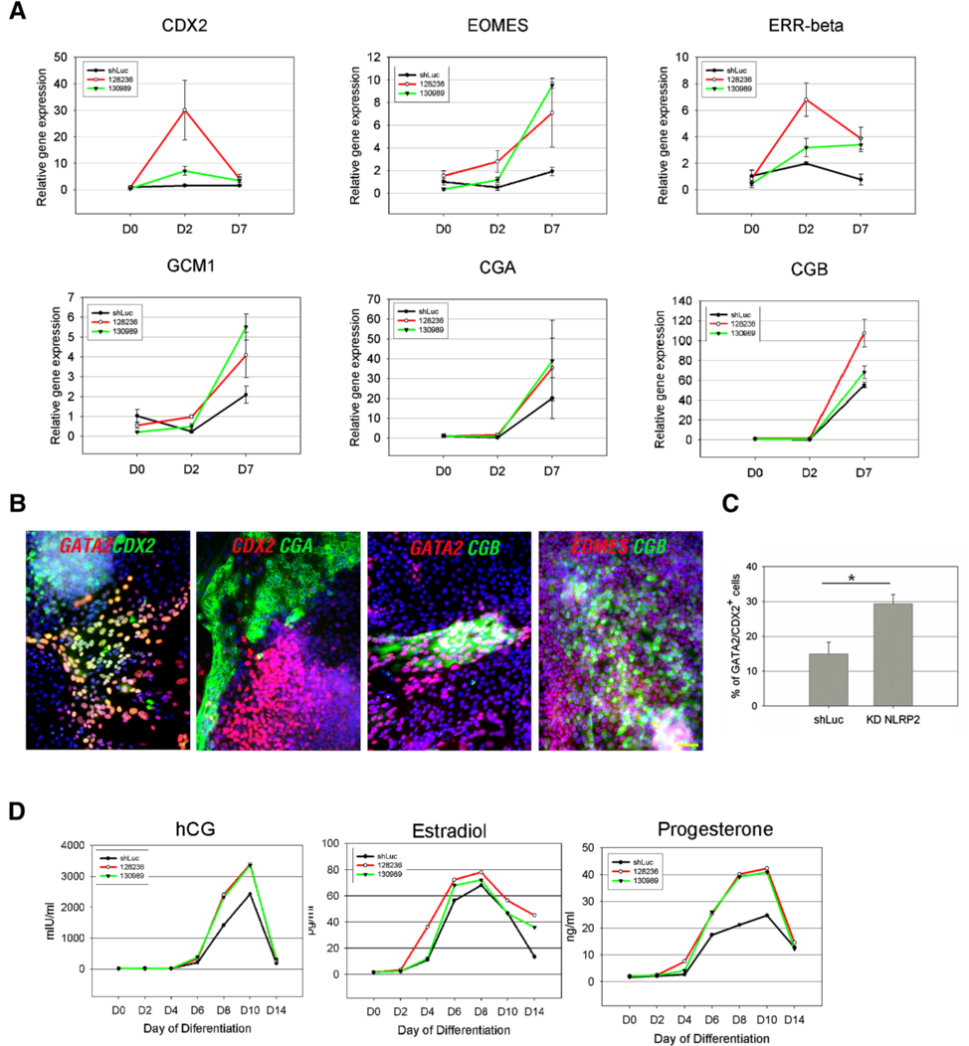
**Trophoblastic differentiation of shLuc and knockdown NLRP2 (128236 and 130989) H9 embryonic stem cells. (A)** Quantitative PCR analysis of the indicated trophoblast markers at days 0 (embryonic stem cell (ESC)), 2, and 7 of differentiation. The relative gene expression was first normalized to that of GAPDH, and then presented as the fold change relative to that in shLuc H9 ESCs. Values are mean ± standard deviation (SD) (*n* = 3). **(B)** Immunofluorescence (IF) staining of trophoblast markers at day 7 of differentiation in 128236 knockdown (KD) NLRP2 human embryonic stem cells (hESCs). Scale bars = 50 μm. **(C)** Quantitative analysis of shLuc and 128236 KD NLRP2 hESCs that exhibited IF staining for both GATA2 and CDX2 at day 7 of differentiation. About 500 to 600 cells were counted for each experiment. Values are mean ± SD (*n* = 3). *P <0.05. **(D)** Immunoassays of three placental hormones (human chorionic gonadotropin (hCG), estradiol, and progesterone) at days 0, 2, 4, 6, 8, 10, and 14 of differentiation.

Next, we examined whether reducing the expression level of NLRP2 influences the *in vitro* differentiation of hESCs. To this end, we subjected hESCs transfected with or without KD NLRP2 to *in vitro* spontaneous differentiation. Quantitative PCR analysis was used to demonstrate that KD NLRP2 upregulated expression of genes associated with trophoblastic lineages, but not other lineages (Additional file 7), consistent with the spontaneous differentiation of iGRAs (Figure 4A). Moreover, treatment of KD NLRP2 hESCs with specific trophoblastic differentiation protocols resulted in higher expression of trophoblast markers (CDX2, EOMES, ERR-β, and GCM1) from differentiation day 2, and CGA and CGB at day 7, as compared with control shLuc hESCs (Figure 7A). IF staining of representative KD NLRP2 hESCs (128236) also revealed protein expression of GATA2, CDX2, EOMES, CGA, and CGB at day 7 of trophoblastic differentiation (Figure 7B). The percentage of cells double positive for GATA2 and CDX2 was about twofold higher for differentiated KD NLRP2 cells than for shLuc cells (29.4% vs. 15%) (Figure 7C). To further confirm that KD NLRP2 hESCs preferentially differentiate into trophoblasts, we measured the amounts of placental hormones (human chorionic gonadotropin, estradiol, and progesterone) in the culture media. We found that concentrations of all three hormones were markedly higher in the media from either type of KD NLRP2 hESCs than in those of control shLuc cells under conditions of trophoblastic differentiation (Figure 7D). Together, these results demonstrate that disruption of NLRP2 expression in hESCs results in a biased differentiation propensity toward trophoblastic lineages in differentiating hESCs.

## Discussion

In this study, we demonstrate that iPSC clones can be generated from human granulosa cells by retroviral transduction of the four transcription factors: SOX2, OCT4, c-MYC, and KLF4 [1]. The established iGRA were similar to hESCs in several respects, including morphological traits, expression of pluripotent markers, and *in vitro* and *in vivo* differentiation propensity. Moreover, the microarray data indicate that the gene transcriptional profiles of iGRAs were much closer to those of hESCs than those of iPSCs derived from other parental sources, such as skin fibroblasts and dermal papilla cells; on the other hand, the expression levels of the NLRP2 gene and the tendency to spontaneously differentiate toward trophoblastic lineages distinguished the iGRAs from hESCs and iPSCs derived from fibroblasts and dermal papilla cells. To confirm the existence of a link between NLRP2 expression and trophoblastic differentiation, we disrupted the expression of NLRP2 in H9 hESCs, and showed that such compromised NLRP2 expression resulted in enhanced trophoblastic differentiation; this finding suggests that NLRP2 may play an important role in modulating trophoblastic differentiation in human pluripotent stem cells. Together, our results demonstrate that human granulosa cells are not only suitable as a source for generating iPSCs, but may also provide a potential model system in which to study trophoblastic differentiation.

We have also demonstrated a new, alternative method for deriving iPSCs from a limited number of parental cells. If cells with different parental origins are mixed, the parental identity of individual iPSC clones can be distinguished by microsatellite assays. In the study described here, we mixed granulosa cells from three individuals, and were able to generate iGRAs from granulosa cells of two different parental origins. However, the majority of the analyzed iGRA lines were derived from one individual (12 of 13 analyzed iPSC lines). Because efficient iPSC generation involves many factors and complex mechanisms, and because the granulosa cells derived from different individuals were all subjected to the same reprogramming conditions in this experiment, we hypothesize that the properties of the parental cell (in terms of genetic, epigenetic, and/or cell growth) may influence iPSC reprogramming. One possible explanation for our finding is thus that the granulosa cells from one individual possessed properties that enabled more efficient growth as compared with those from the other two individuals. Nevertheless, the demonstrated one-pot method for iPSC derivation not only enables the simultaneous derivation of iPSC lines from a small number of somatic cells from multiple individuals, but also decreases the time and effort required for such derivation.

Human granulosa cells have been reported to be multipotent in the presence of leukemia inhibitory factor [12]. It also has been reported that certain granulosa cells express OCT4 mRNA, which has been attributed to their association with oocytes during gamete formation and maturation [20]. It is believed that oocytes contain a powerful reprogramming factor, which enables the totipotency reprogramming of differentiated somatic nuclei. This collection of unique characteristics has made granulosa cells particularly suitable as a parental cell population for somatic cell nuclear transfer in animals [21]. Recently, Mao and colleagues also showed that mouse iPSCs could be efficiently generated from granulosa cells using only Oct4 and Sox2 [22]. Therefore, it is tempting to use granulosa cells as parental cells for efficient hiPSC derivation. However, we found that the efficiency of iGRA generation is not significantly higher than that of foreskin-derived or follicle dermal papilla cell-derived iPSCs, in terms of the numbers of either early reprogramming colonies or reprogrammed iPSC lines (Table 1). This may be explained by the fact that our RT-PCR analysis did not detect OCT4 expression in the granulosa cells used for iGRA derivation. Although the derivation efficiency of iGRAs does not exceed that of other somatic cell-derived iPSCs, the microarray data indicated that the global gene expression pattern of iGRAs was much closer to that of hESCs (H9 and NTU1) than those of other iPSC lines. Whether this phenomenon can be attributed to the aforementioned unique characteristics of granulosa cells will require further investigation.

We consistently observed that expression levels of the NLRP2 gene are lower in iGRAs than in iPSCs derived from other somatic cells, and that iGRAs have a tendency to spontaneously differentiate toward a trophoblastic fate. NLRP2, a member of the NLRP (nucleotide-binding oligomerization domain, leucine rich repeat and pyrin domain containing) family, was recently shown to be required for both human and mouse early embryonic development [18,19]. In mice, KD of NLRP2 in oocytes and zygotes dramatically compromised developmental competence [18]. In addition, mutations of NLRP2 have been discovered to associate with the human imprinting disorder Beckwith–Wiedemann syndrome, which causes fetal overgrowth; in addition, a second study suggested an association between NLRP2 and recurrent miscarriages [23,24]. Since our granulosa parental cells were obtained from patients undergoing infertility treatment, there is a possibility that the granulosa cells carried genetic or epigenetic defects in the NLRP2 gene, thereby resulting in lower expression of NLRP2 in the iGRAs. Failure to erase parental memory during iPSC reprogramming is also a possible reason for the low expression of NLRP2 in iGRAs.

NLRP family genes are well known for their role in apoptosis and inflammation [25,26]; several members of the NLRP family, including NLRP2, have been shown to inhibit the nuclear factor (NF)-κB signaling pathway, and function as a modulator of the inflammatory response [27]. In mouse ESCs, increased expression of NF-κB signaling has been implicated in ESC differentiation [28,29], and it also has been demonstrated that Nanog maintains mESC pluripotency through inhibition of NF-κB activity [29]. The role of NF-κB signaling in the maintenance of human pluripotency is still a matter of debate. Augmentation of NF-κB signaling has been suggested to maintain the undifferentiated status of human pluripotent stem cells [30,31]. However, Yang and colleagues showed that an increase of canonical NF-κB signaling was associated with differentiation of hESCs [32]. Here, we show that KD of NLRP2 did not result in either downregulation of NF-κB signaling or altered pluripotency/differentiation of hESCs when compared with wildtype hESCs (Additional file 8). Therefore, our data support the hypothesis that NLRP2 may not directly regulate pluripotency maintenance via NF-κB signaling in hESCs. The role of NLRP2 in undifferentiated hESCs, and how reduced expression of NLRP2 results in preferential differentiation of hESCs toward the trophoblastic lineage, remain to be clarified.

Although there is currently no clear evidence implicating NLRP2 in trophoblast development, mutations of NLRP7 have been reported to cause recurrent hydatidiform moles, an abnormality of pregnancy that is characterized by hypertrophic vesicular trophoblasts in human [33-35]. Recently, Mahadevan and colleagues demonstrated that reduced expression of NLRP7 altered DNA methylation and accelerated trophoblastic lineage differentiation in hESC cultures [36]. Interesting, NLRP2 was found to possess a similar function to that of NLRP7. No ortholog of human NLRP7 is present in the mouse genome; rather, mouse NLRP7 is believed to have arisen from mouse NLRP2 by a gene duplication event [34,37]. Therefore, it is tempting to suggest that NLRP2 and NLRP7 may share a redundant role in regulating trophoblastic development, although the mechanism by which NLRP2 regulates trophoblast development is currently unknown.

## Conclusions

In summary, we have demonstrated a new method of simultaneously generating iPSC clones from mixed granulosa cell populations derived from multiple individuals. While the iGRAs resemble hESCs in several ways, their NLRP2 expression levels were generally lower than those of hESCs and iPSCs derived from other parental origins. By comparing the differentiation potentials of iGRAs, hESCS, and other iPSC lines, we found that iGRAs exhibit preferential differentiation towards the trophoblastic lineage. Importantly, KD of NLRP2 in hESCs also results in the promotion of trophoblastic differentiation *in vitro*. Together, these results suggest that NLRP2 may play a role in modulating trophoblastic differentiation, although the mechanisms involved require further elucidation.

## Additional files

Additional file 1:
**Primer sets used for (A) RT-PCR and (B) quantitative PCR.**
Additional file 2:
**Is a figure showing the microsatellite marker analysis for human iGRAs.**
Additional file 3:
**Is a table presenting a summary of the correlation of gene expression in H9, iCFB46, iCFB50, and iGRA1, iGRA2 and iGRA7.** The relative gene expression of (A) mesoderm, (B) ectoderm, (C) endoderm, (D) germ cells and (E) trophoblasts. P <0.05 labeled in red, P <0.01 labeled in blue, P <0.001 labeled in purple. Additional file 4:
**Is a figure showing the hierarchical clustering of microarray-based gene expression profiles from multiple hESCs, hiPSCs, and their parental cells.** DPC, human follicle dermal papilla cells; Fore, foreskin cells; Gra, granulosa cells. Additional file 5:
**Is a figure showing the gene expression of NLRP2, NLRP7, and NLRP12 in hESCs, iPSCs, and their corresponding parental cells.** **(A)** Expression patterns as determined using microarray data. **(B)** Expression patterns as determined using quantitative PCR. The relative gene expression levels were normalized to those in H9 hESCs. Additional file 6:
**Is a figure showing the gene expression of NLRP2, NLRP7, and NLRP12 before and during** ***in vitro*** **differentiation (day 20) in hESCs (H9 and NTU1) and iPSCs (iCFB10 and iCFB46, iDPC4 and iGRA1), as determined by RT-PCR.** UD, undifferentiated hESCs or iPSCs; D, differentiated hESCs or iPSCs; N, negative control for RT-PCR. Additional file 7:
**Is a figure showing the quantitative PCR analysis of genes related to pluripotency, germ layers (ectoderm, mesoderm, and endoderm), germline, and trophoblast in** ***in vitro*** **differentiated shLuc and KD NLRP2 (128236 and 130989) H9 ESCs at days 10 and 20 of differentiation.** Relative gene expression was first normalized to that of GAPDH, and then presented as the fold change relative to H9. Values are mean ± standard deviation (*n* = 3). Black line, shLuc H9 ESCs; red line, KD NLRP2 (128236) H9 ESCs; green line, KD NLRP2 (130989) H9 ESCs. Additional file 8:
**Is a figure showing the quantitative PCR analysis of genes related to NF-κB signaling in** ***in vitro*** **differentiated shLuc and KD NLRP2 (128236 and 130989) H9 ESCs at days 10 and 20 of differentiation. Relative gene expression was first normalized to that of GAPDH, and then presented as the fold change relative to shLuc H9 ESCs.** Values are mean ± standard deviation (*n* = 3). ESC, undifferentiated ESCs; RD10, *in vitro* random differentiation day 10; RD20, *in vitro* random differentiation day 20. Black line, shLuc H9 ESCs; red line, KD NLRP2 (128236) H9 ESCs; green line, KD NLRP2 (130989) H9 ESCs.

## Abbreviations

ESC: embryonic stem cell
hESC: human embryonic stem cell
hiPSC: human induced pluripotent stem cell
IF: immunofluorescence
iGRA: granulosa cell-derived induced pluripotent stem cell
iPSC: induced pluripotent stem cell
KD: knockdown
NF: nuclear factor
NLRP: nucleotide-binding oligomerization domain, leucine rich repeat and pyrin domain containing
