# Supplementary material for: Granulosa cell-derived induced pluripotent stem cells exhibit pro-trophoblastic differentiation potential
Source: Stem Cell Res Ther. 2015 Feb 27;6(1):14. doi: 10.1186/s13287-015-0005-5 (PMC4430911; doi:10.1186/s13287-015-0005-5)
Supplement: Additional file 1: — Primer sets used for (A) RT-PCR and (B) quantitative PCR. [file 13287_2015_5_MOESM1_ESM.docx]

**Supplementary table I:**

1. Primer sets used for RT-PCR

| Primer Name | Sequence (5′ to 3′) |
| --- | --- |
| Hand1-F | TGCCTGAGAAAGAGAACCAG |
| Hand1-r | ATGGCAGGATGAACAAACAC |
| cTNI-F | TCCTCCAACTACCGCGCTTA |
| cTNI-R | CTTCATCCACCTTGTCCACA |
| GATA4-F | CTACAGGGGCACTTAACCCA |
| GATA4-R | AGAGCTGAATCGCTCAGAGC |
| PAX6-F | CCGAGAGTAGCGACTCCAG |
| PAX6-R | CTTCCGGTCTGCCCGTTC |
| SOX1-F | AGTTTAATGAGAACCGAATTCAGC |
| SOX1-R | TGTAATCACTTTAACGAATGAGTGG |
| MAP2-F | GCATGAGCTCTTGGCAGG |
| MAP2-R | CCAATTGAACCCATGTAAAGCC |
| GATA6-F | CCTCACTCCACTCGTGTCTGC |
| GATA6-R | GTCCTGGCTTCTGGAAGTGG |
| AFP-F | AAATACATCCAGGAGAGCCA |
| AFP-R | CTGAGCTTGGCACAGATCCT |
| HNF4A-F | CCCAGCCCCCTAAGAGAGCAC |
| HNF4A-R | GGATGAAGGTGAAGGTGAAGG |

1. Primer sets used for quantitative PCR

| Primer Name | Sequence (5′ to 3′) |
| --- | --- |
| OCT4-F | TGGGCTCGAGAAGGATGTG |
| OCT4-R | CTTGATCGCTTGCCCTTCTG |
| NANOG-F | CAGCAGATGCAAGAACTCTCCA |
| NANOG-R | CATTGCTATTCTTCGGCCAGT |
| SOX2-F | CATCACCCACAGCAAATGAC |
| SOX2-R | CAAACTTCCTGCAAAGCTCC |
| NLRP2-F | GATGATATGTTTCCCGCATTG |
| NLRP2-R | GAGAGGTTTACGCACGTC |
| NLRP7-F | CATCCTAGCCCAAGCACA |
| NLRP7-R | ATCTTCCTCTTCAGCAAACTC |
| NLRP12-F | ATGATTCAGTTGAGGAAGTGT |
| NLRP12-R | CCTCAGTCCCTGGCATAGTAA |
| NANOS3-F | GCAAGGGAAGAGCTGAAATCG |
| NANOS3-R | AGCACTAGGGAAACGGCAGAT |
| STELLA-F | CGGGCTACCTGGTAGCAATTT |
| STELLA-R | TCCAGGATCTTTGAAACGTAGCA |
| VASA-F | TCTTCCTTCTACCATTGATGAATATGTT |
| VASA-R | CTGCCAGTATTCCCACAACGA |
| MIXL1-F | AACGAAATGTCTGAAGCCCCA |
| MIXL1-R | TCCTCCCATGAGTCCAGCTTT |
| BRACHYURY(T)-F | CCAATGAGATGATCGTGACCA |
| BRACHYURY(T)-R | ATTCCCCGTTCACGTACTTCC |
| cTNI-F | GGAGGACACCGAGAAGGA AAAC |
| cTNI-R | TTCCAC TCAGTGCATCGATGTT |
| HAND1-F | TACCTGATGGACGTGCTGG |
| HAND1-R | CCTCGGCTCACTGGTTTA |
| PAX6-F | GATAACATACCAAGCGTGTCATCAATA |
| PAX6-R | TGCGCCCATCTGTTGC |
| SOX1-F | TGGCATCTAGGTCTTGGCTCA |
| SOX1-R | GCACGAAGCACCTGCAATAAG |
| MAP2-F | GCTCTGGCTCCCAGTGTATTTAA |
| MAP2-R | CCTGTAAAGCAGGAATCTTTGACA |
| SOX17-F | TTCGTGTGCAAGCCTGAGAT |
| SOX17-R | GTGTGTAACACTGCTTCTGGCC |
| GATA6-F | ACTAACCCACAGGCAGGTTG |
| GATA6-R | GGTACAAAACGGCTCCAAAA |
| AFP-F | CAAGTTCCAGAACCTGTCACAA |
| AFP-R | CAGCCCAAAGAAGAATTGTAG |
| HNF4A-F | GCTGCAGATCGATGACAATGA |
| HNF4A-R | CTTGGCATCTGGGTCAAAGAA G |
| CDX2-F | GTTGCTGCTGTTTGGGTTGTT |
| CDX2-R | CATGGA TCCAGA AGGCTTTAA AA |
| EOMES-F | CGGAGCCCTTTGTCA ACACT |
| EOMES-R | TGGTCTGTGGCACGGTTCT |
| ERRβ-F | CAGCTGCACCTCACTGGATCT |
| ERRβ-R | TTCCGCCCCACTCTGCTA |
| GCM1-F | CTCTGAAGCTCATCCCTTGCC |
| GCM1-R | TGGACGCCTTCCTGGAAAGAC |
| CGA-F | CAACCGCCCTGAACACATCC |
| CGA-R | CAGCAAGTGGACTCTGAGGTG |
| CGB-F | TGAGATCACTTCACCGTGGTCTCC |
| CGB-R | TTTATACCTCGGGGTTGTGGGG |
| GAPDH-F | CGGGAAACTGTGGCGTGATG |
| GAPDH-R | TG TGGAGGAGTGGGTGTCGCTGTT |
