# Supplementary figures and images for: Granulosa cell-derived induced pluripotent stem cells exhibit pro-trophoblastic differentiation potential
Source: Stem Cell Res Ther. 2015 Feb 27;6(1):14. doi: 10.1186/s13287-015-0005-5 (PMC4430911; doi:10.1186/s13287-015-0005-5)

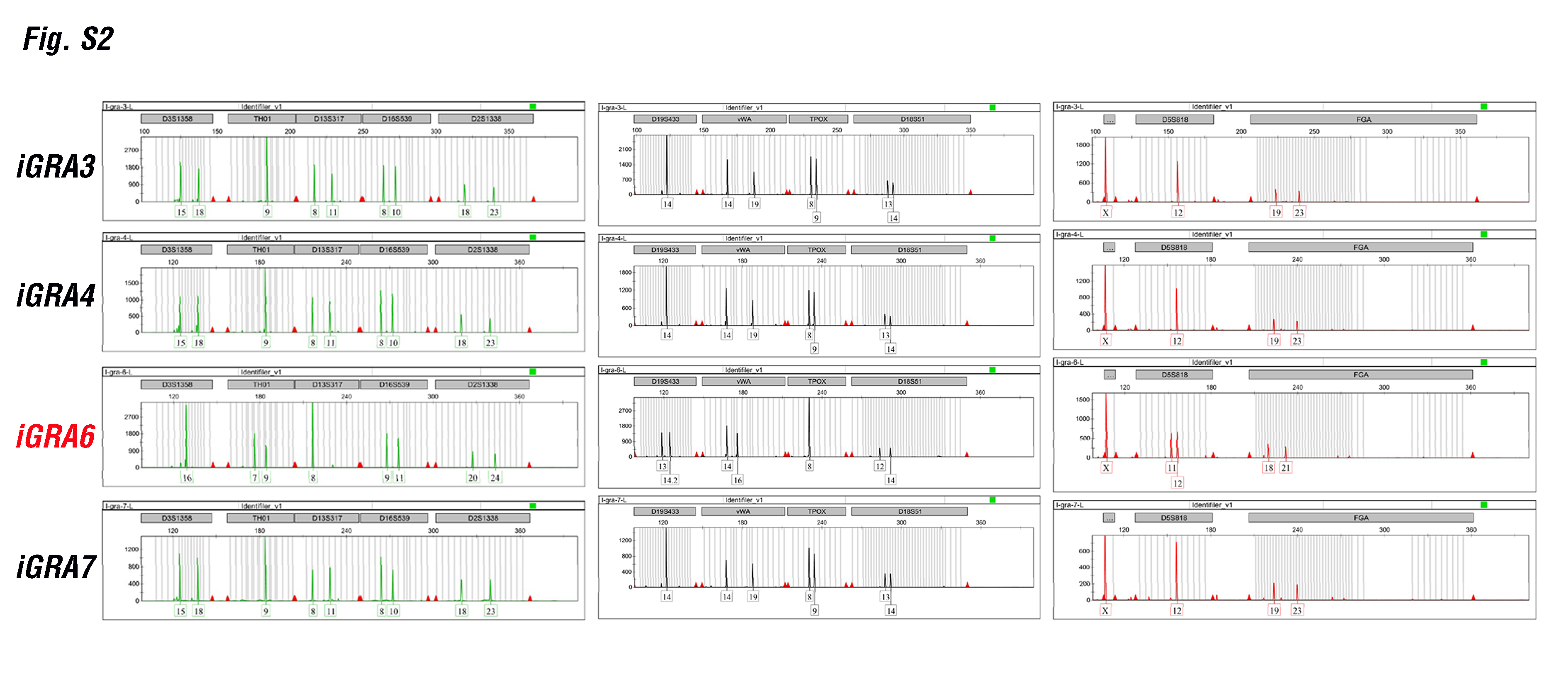

Supplement: Additional file 2: — Is a figure showing the microsatellite marker analysis for human iGRAs. [file 13287_2015_5_MOESM2_ESM.tiff]

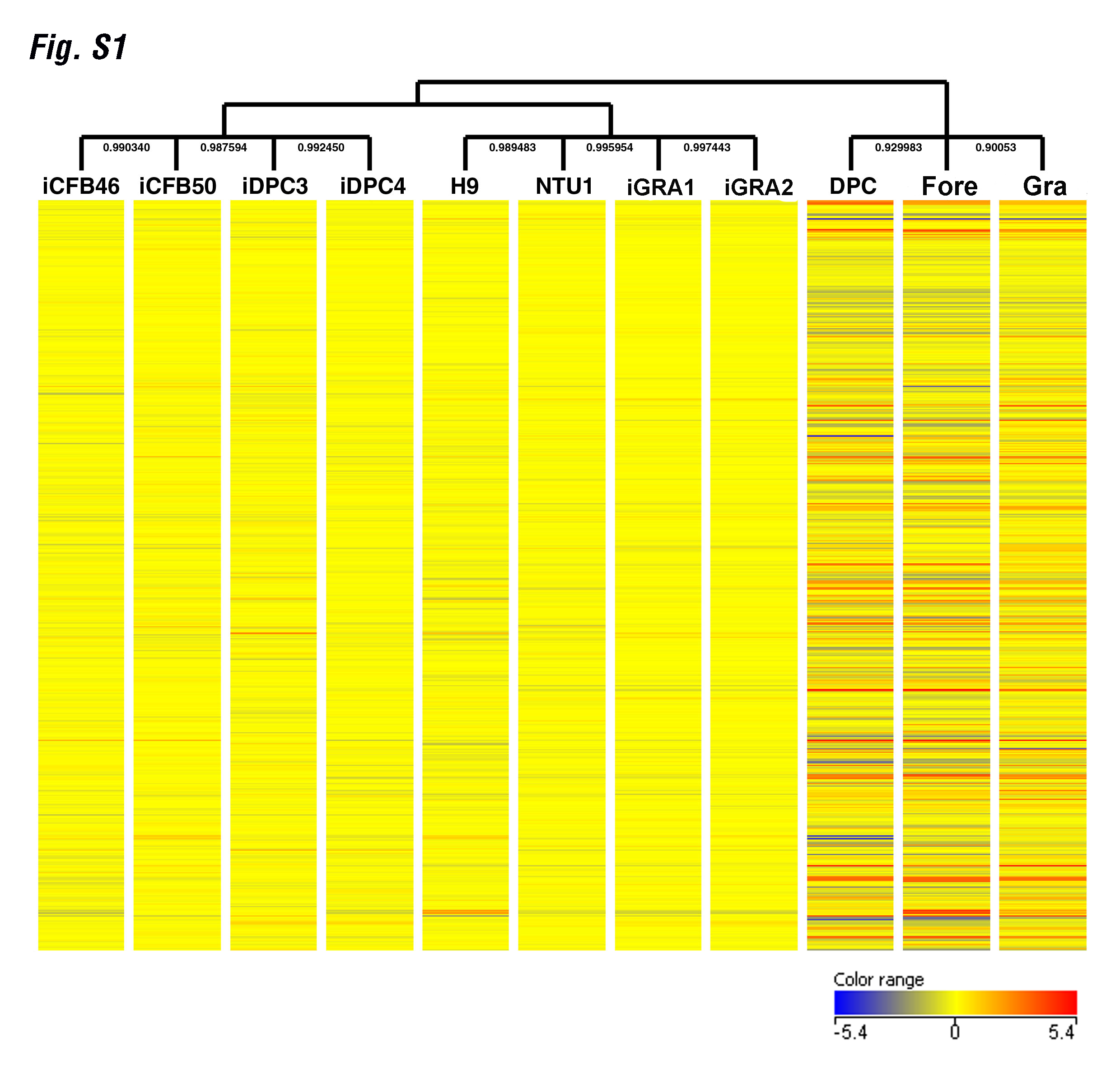

Supplement: Additional file 4: — Is a figure showing the hierarchical clustering of microarray-based gene expression profiles from multiple hESCs, hiPSCs, and their parental cells. DPC, human follicle dermal papilla cells; Fore, foreskin cells; Gra, granulosa cells. [file 13287_2015_5_MOESM4_ESM.tiff]

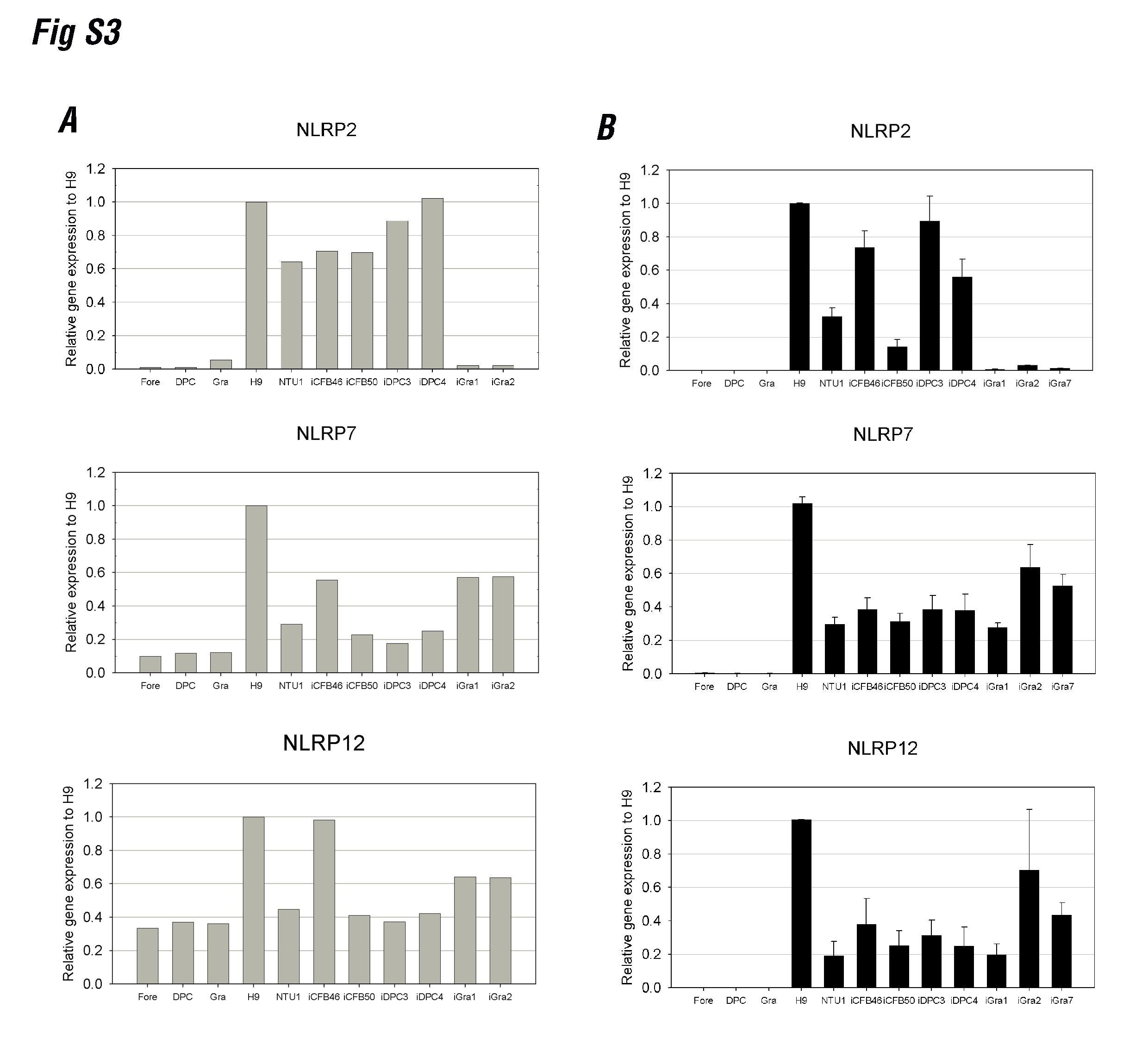

Supplement: Additional file 5: — Is a figure showing the gene expression of NLRP2, NLRP7, and NLRP12 in hESCs, iPSCs, and their corresponding parental cells. (A) Expression patterns as determined using microarray data. (B) Expression patterns as determined using quantitative PCR. The relative gene expression levels were normalized to those in H9 hESCs. [file 13287_2015_5_MOESM5_ESM.tiff]

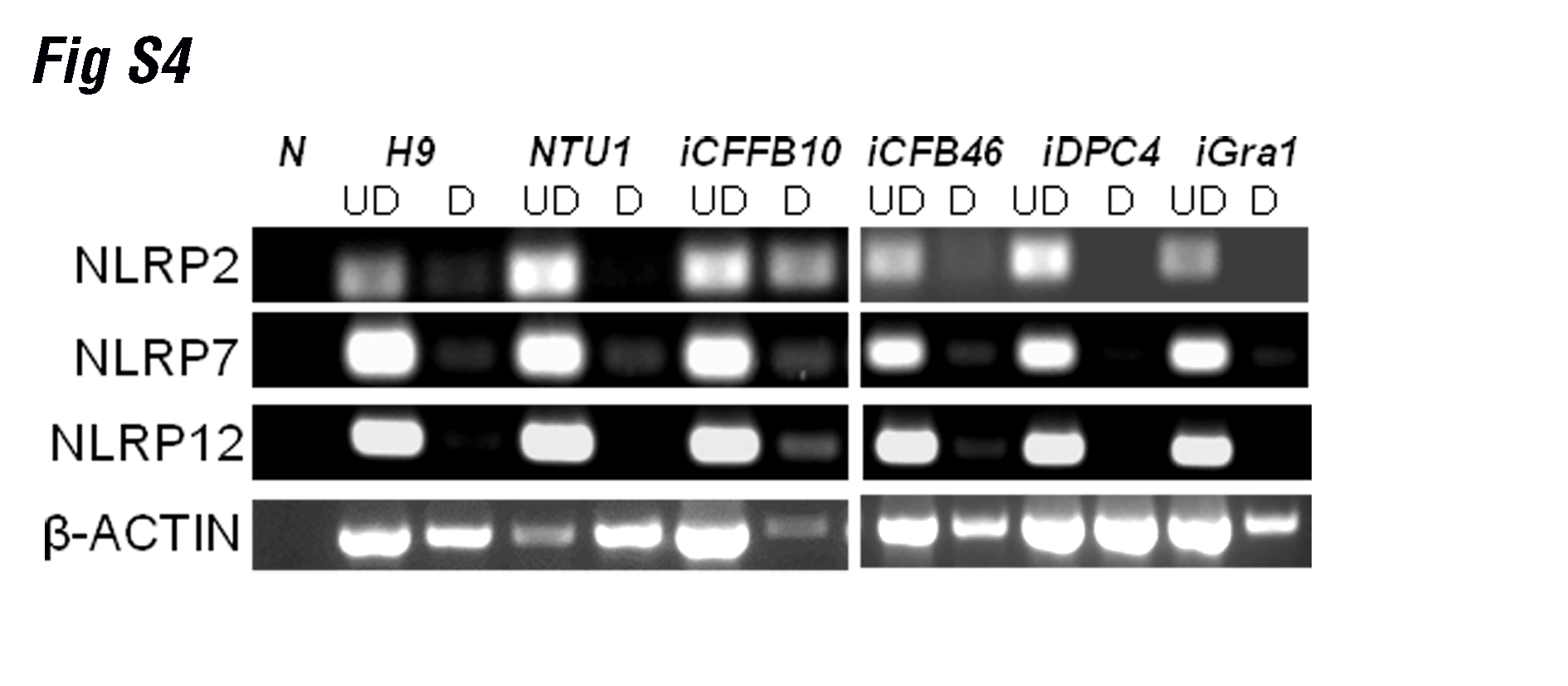

Supplement: Additional file 6: — Is a figure showing the gene expression of NLRP2, NLRP7, and NLRP12 before and during in vitro differentiation (day 20) in hESCs (H9 and NTU1) and iPSCs (iCFB10 and iCFB46, iDPC4 and iGRA1), as determined by RT-PCR. UD, undifferentiated hESCs or iPSCs; D, differentiated hESCs or iPSCs; N, negative control for RT-PCR. [file 13287_2015_5_MOESM6_ESM.tiff]

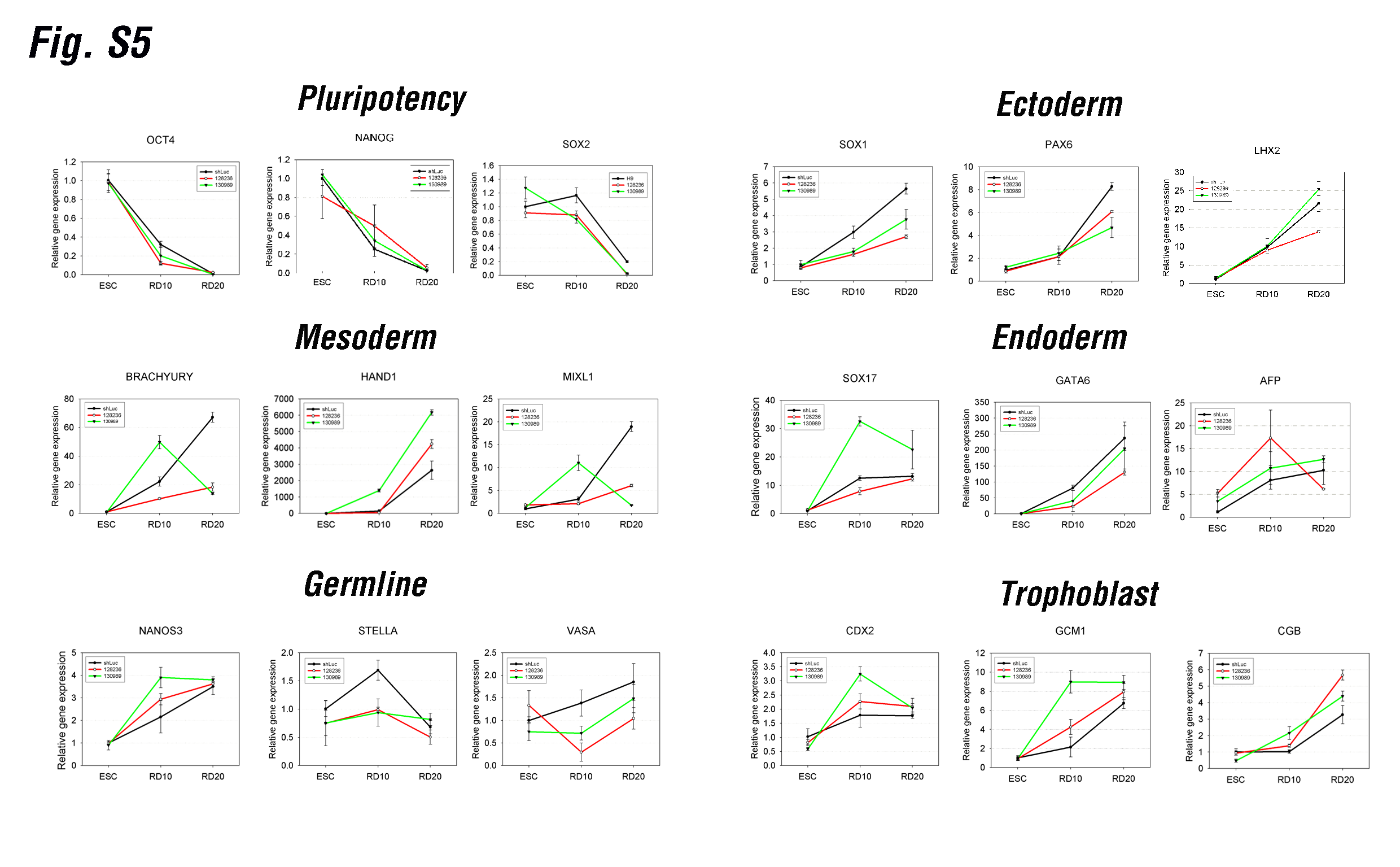

Supplement: Additional file 7: — Is a figure showing the quantitative PCR analysis of genes related to pluripotency, germ layers (ectoderm, mesoderm, and endoderm), germline, and trophoblast in in vitro differentiated shLuc and KD NLRP2 (128236 and 130989) H9 ESCs at days 10 and 20 of differentiation. Relative gene expression was first normalized to that of GAPDH, and then presented as the fold change relative to H9. Values are mean ± standard deviation (n = 3). Black line, shLuc H9 ESCs; red line, KD NLRP2 (128236) H9 ESCs; green line, KD NLRP2 (130989) H9 ESCs. [file 13287_2015_5_MOESM7_ESM.tiff]

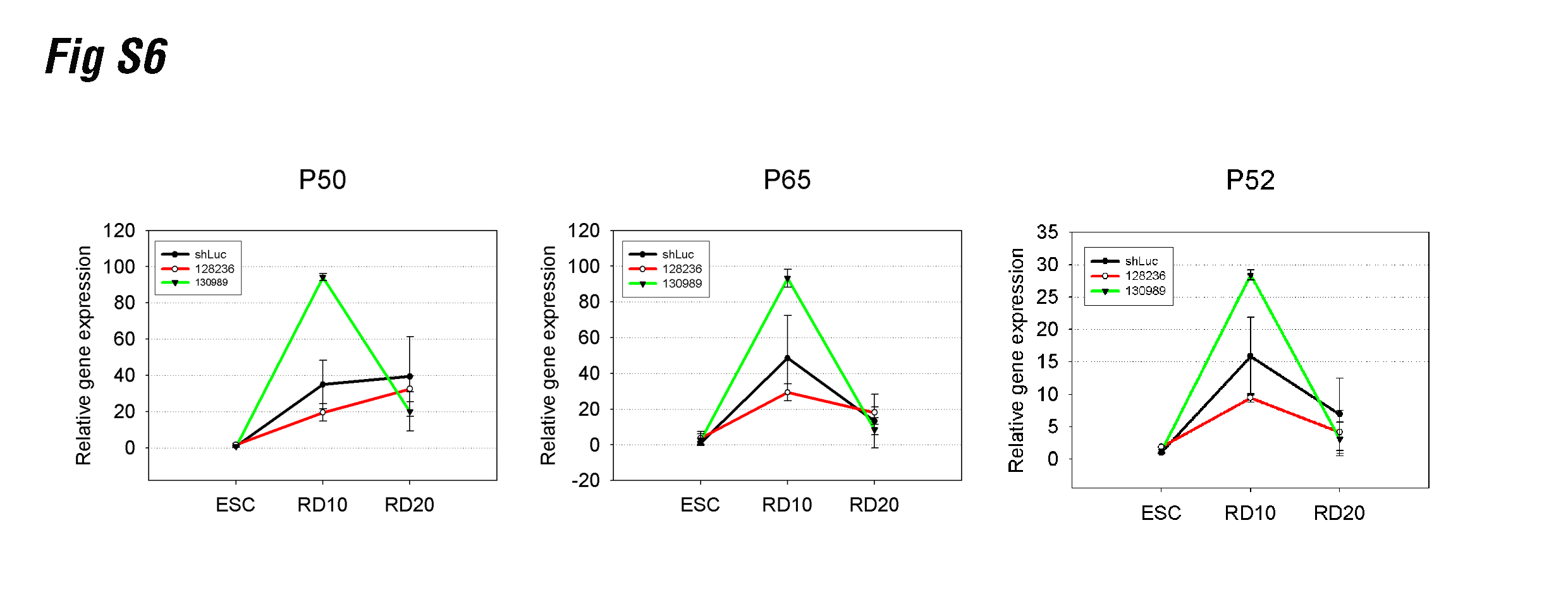

Supplement: Additional file 8: — Is a figure showing the quantitative PCR analysis of genes related to NF-κB signaling in in vitro differentiated shLuc and KD NLRP2 (128236 and 130989) H9 ESCs at days 10 and 20 of differentiation. Relative gene expression was first normalized to that of GAPDH, and then presented as the fold change relative to shLuc H9 ESCs. Values are mean ± standard deviation (n = 3). ESC, undifferentiated ESCs; RD10, in vitro random differentiation day 10; RD20, in vitro random differentiation day 20. Black line, shLuc H9 ESCs; red line, KD NLRP2 (128236) H9 ESCs; green line, KD NLRP2 (130989) H9 ESCs. [file 13287_2015_5_MOESM8_ESM.tiff]
