# Supplementary material for: Granulosa cell-derived induced pluripotent stem cells exhibit pro-trophoblastic differentiation potential
Source: Stem Cell Res Ther. 2015 Feb 27;6(1):14. doi: 10.1186/s13287-015-0005-5 (PMC4430911; doi:10.1186/s13287-015-0005-5)
Supplement: Additional file 3: — Is a table presenting a summary of the correlation of gene expression in H9, iCFB46, iCFB50, and iGRA1, iGRA2 and iGRA7. The relative gene expression of (A) mesoderm, (B) ectoderm, (C) endoderm, (D) germ cells and (E) trophoblasts. P <0.05 labeled in red, P <0.01 labeled in blue, P <0.001 labeled in purple. [file 13287_2015_5_MOESM3_ESM.docx]

**Supplementary Table II**

Summary of the correlation of gene expression in H9, iCFB46, iCFB50, and iGRA1, 2 and 7. The relative gene expression of (A) mesoderm, (B) ectoderm, (C) endoderm, (D) germ cells and (E) trophoblasts. P < 0.05 labeled in red, p < 0.01 labeled in blue, p < 0.001 labeled in purple.

**A. Mesoderm**

**a. BRA**

|  | iGRA1 | iGRA2 | iGRA7 |
| --- | --- | --- | --- |
| H9 | 0.209067 | 0.242715 | 0.386489 |
| iCFB46 | 0.030212 | 0.243549 | 0.243549 |
| iCFB50 | 0.028744 | 0.068649 | 0.13202 |

**b. MIXL1**

|  | iGRA1 | iGRA2 | iGRA7 |
| --- | --- | --- | --- |
| H9 | 0.027582 | 0.134168 | 0.218055 |
| iCFB46 | 0.011319 | 0.011556 | 0.347269 |
| iCFB50 | 0.139491 | 0.139752 | 0.162131 |

**c. cTnI**

|  | iGRA1 | iGRA2 | iGRA7 |
| --- | --- | --- | --- |
| H9 | 0.108477 | 0.200543 | 0.273114 |
| iCFB46 | 0.048392 | 0.294441 | 0.094347 |
| iCFB50 | 0.126574 | 0.169253 | 0.062481 |

**B. Ectoderm**

**a. SOX1**

|  | iGRA1 | iGRA2 | iGRA7 |
| --- | --- | --- | --- |
| H9 | 0.283975 | 0.128616 | 0.120694 |
| iCFB46 | 0.008811 | 0.024953 | 0.059776 |
| iCFB50 | 0.193793 | 0.124706 | 0.118904 |

**b. PAX6**

|  | iGRA1 | iGRA2 | iGRA7 |
| --- | --- | --- | --- |
| H9 | 0.137665 | 0.07366 | 0.068762 |
| iCFB46 | 0.056937 | 0.088565 | 0.271031 |
| iCFB50 | 0.080953 | 0.020598 | 0.029211 |

**c. MAP2**

|  | iGRA1 | iGRA2 | iGRA7 |
| --- | --- | --- | --- |
| H9 | 0.056573 | 0.007763 | 0.216497 |
| iCFB46 | 0.01105 | 0.072777 | 0.10916 |
| iCFB50 | 0.115436 | 0.465862 | 0.409073 |

**C. Endoderm**

**a. SOX17**

|  | iGRA1 | iGRA2 | iGRA7 |
| --- | --- | --- | --- |
| H9 | 0.004747 | 0.166456 | 0.184376 |
| iCFB46 | 0.015071 | 0.003011 | 0.139944 |
| iCFB50 | 0.132185 | 0.410778 | 0.307475 |

**b. AFP**

|  | iGRA1 | iGRA2 | iGRA7 |
| --- | --- | --- | --- |
| H9 | 0.103187 | 0.420012 | 0.069038 |
| iCFB46 | 0.287665 | 0.420247 | 0.122206 |
| iCFB50 | 0.196306 | 0.409525 | 0.056718 |

**c. HNF4A**

|  | iGRA1 | iGRA2 | iGRA7 |
| --- | --- | --- | --- |
| H9 | 0.305102 | 0.404591 | 0.338455 |
| iCFB46 | 0.457656 | 0.276748 | 0.415447 |
| iCFB50 | 0.253349 | 0.242485 | 0.241778 |

**D. Germ cells**

**a. NANOS3**

|  | iGRA1 | iGRA2 | iGRA7 |
| --- | --- | --- | --- |
| H9 | 0.010087 | 0.133436 | 0.059576 |
| iCFB46 | 0.008089 | 0.10472 | 0.054612 |
| iCFB50 | 0.007532 | 0.106769 | 0.055389 |

**b. STELLA**

|  | iGRA1 | iGRA2 | iGRA7 |
| --- | --- | --- | --- |
| H9 | 0.000367 | 0.286446 | 0.157868 |
| iCFB46 | 0.012815 | 0.10472 | 0.142892 |
| iCFB50 | 0.238538 | 0.335562 | 0.335952 |

**c. VASA**

|  | iGRA1 | iGRA2 | iGRA7 |
| --- | --- | --- | --- |
| H9 | 0.016558 | 0.359831 | 0.404857 |
| iCFB46 | 0.009707 | 0.110397 | 0.135477 |
| iCFB50 | 0.09194 | 0.192386 | 0.189175 |

**E. Trophoblasts**

**a. CDX2**

|  | iGRA1 | iGRA2 | iGRA7 |
| --- | --- | --- | --- |
| H9 | 0.056132 | 0.040194 | 0.072035 |
| iCFB46 | 0.027896 | 0.021819 | 0.026371 |
| iCFB50 | 0.042167 | 0.004569 | 0.029643 |

**b. EOMES**

|  | iGRA1 | iGRA2 | iGRA7 |
| --- | --- | --- | --- |
| H9 | 0.049027 | 0.038274 | 0.211874 |
| iCFB46 | 0.043537 | 0.033343 | 0.091006 |
| iCFB50 | 0.049027 | 0.005459 | 0.064804 |

**c. ERRB**

|  | iGRA1 | iGRA2 | iGRA7 |
| --- | --- | --- | --- |
| H9 | 0.040565 | 0.057258 | 0.02754 |
| iCFB46 | 0.030449 | 0.037831 | 0.025111 |
| iCFB50 | 0.032446 | 0.040979 | 0.02522 |
